# Systemic Effects Induced by Hyperoxia in a Preclinical Model of Intra-abdominal Sepsis

**DOI:** 10.1155/2020/5101834

**Published:** 2020-10-15

**Authors:** M. Isabel García-Laorden, Raquel Rodríguez-González, José L. Martín-Barrasa, Sonia García-Hernández, Ángela Ramos-Nuez, H. Celeste González-García, Jesús M. González-Martín, Robert M. Kacmarek, Jesús Villar

**Affiliations:** ^1^CIBER de Enfermedades Respiratorias, Instituto de Salud Carlos III, Monforte de Lemos 3-5, Pabellón 11, 28029 Madrid, Spain; ^2^Multidisciplinary Organ Dysfunction Evaluation Research Network, Research Unit, Hospital Universitario de Gran Canaria Dr. Negrín, Barranco de la Ballena s/n, 35019 Las Palmas de Gran Canaria, Spain; ^3^Department of Psychiatry, Radiology, Public Health, Nursing and Medicine, School of Nursing, University of Santiago de Compostela, Avda. Xoán XXIII s/n, 15782 Santiago de Compostela, Spain; ^4^Department of Anaesthesiology, Critical Care and Pain Management, Hospital Clínico Universitario, Health Research Institute of Santiago de Compostela (IDIS), Travesa da Choupana s/n, 15706 Santiago de Compostela, Spain; ^5^Animal Infectious Diseases and Ictiopathology, Universitary Research Institute for Terrestrial and Aquatic Animal Health and Food Safety, University of Las Palmas de Gran Canaria, Carretera de Trasmontaña s/n, 35416 Arucas, Spain; ^6^Department of Pathology, Hospital Universitario de Canarias, Carretera Cuesta Taco 0, 38320 Sta. Cruz de Tenerife, Spain; ^7^Department of Respiratory Care, Massachusetts General Hospital, 55 Fruit Street, Boston, MA 02114, USA; ^8^Department of Anaesthesiology, Harvard University, 55 Fruit Street, Boston, MA 02114, USA; ^9^Keenan Research Center for Biomedical Science at the Li Ka Shing Knowledge Institute, St. Michael's Hospital, 209 Victoria Street, M5B1T8, Toronto, ON, Canada

## Abstract

Supplemental oxygen is a supportive treatment in patients with sepsis to balance tissue oxygen delivery and demand in the tissues. However, hyperoxia may induce some pathological effects. We sought to assess organ damage associated with hyperoxia and its correlation with the production of reactive oxygen species (ROS) in a preclinical model of intra-abdominal sepsis. For this purpose, sepsis was induced in male, Sprague-Dawley rats by cecal ligation and puncture (CLP). We randomly assigned experimental animals to three groups: control (healthy animals), septic (CLP), and sham-septic (surgical intervention without CLP). At 18 h after CLP, septic (*n* = 39), sham-septic (*n* = 16), and healthy (*n* = 24) animals were placed within a sealed Plexiglas cage and randomly distributed into four groups for continuous treatment with 21%, 40%, 60%, or 100% oxygen for 24 h. At the end of the experimental period, we evaluated serum levels of cytokines, organ damage biomarkers, histological examination of brain and lung tissue, and ROS production in each surviving animal. We found that high oxygen concentrations increased IL-6 and biomarkers of organ damage levels in septic animals, although no relevant histopathological lung or brain damage was observed. Healthy rats had an increase in IL-6 and aspartate aminotransferase at high oxygen concentration. IL-6 levels, but not ROS levels, are correlated with markers of organ damage. In our study, the use of high oxygen concentrations in a clinically relevant model of intra-abdominal sepsis was associated with enhanced inflammation and organ damage. These findings were unrelated to ROS release into circulation. Hyperoxia could exacerbate sepsis-induced inflammation, and it could be by itself detrimental. Our study highlights the need of developing safer thresholds for oxygen therapy.

## 1. Introduction

Sepsis is a life-threatening organ dysfunction syndrome that results from a deregulated host response to infection [1]. Sepsis remains a main cause of hospital mortality, and it is also associated with poor long-term outcomes after hospital discharge [1–3]. Organ system failures defining sepsis include circulatory, renal, pulmonary, hepatic, hematologic, and central nervous systems [4]. Multiple organ dysfunction (MOD) is more common than single organ failure during sepsis. Sepsis is the most common cause of acute respiratory failure in critically ill patients, and the respiratory system dysfunction is mainly characterized by hypoxemia.

Supplemental oxygen (O_2_) is a common therapy administered to septic patients to balance tissue oxygen delivery and demand. Previous studies had reported beneficial effects of hyperoxia in sepsis and in MOD. Studies in experimental models have shown that hyperoxia plays a role in the regulation of inflammatory cytokines and antioxidants, attenuation of apoptosis, improvement of organ function, and antibiotic action [5–11]. However, while oxygen therapy can be lifesaving, it can also induce pathological effects. The hyperoxic environment created by supraphysiological concentrations of O_2_ may induce an increase in the production of reactive oxygen species (ROS). ROS cause lipid peroxidation, protein oxidation, and DNA breakage [12]. When the generation of ROS is higher than the capacity of the system to neutralize and eliminate them, oxidative stress, cell death, inflammation, and modulation of cell growth may occur [13, 14], leading to tissue damage and organ injury. Although harmful effects of hyperoxia have been described in experimental studies in rodent models and in critically ill patients [15–25], hyperoxia-induced organ injury is still an open field for intense research.

We have previously reported deleterious effects of hyperoxia in a rodent model of sepsis by cecal ligation and puncture (CLP), followed by a 24-hour exposure to different O_2_ concentrations 18 hours after CLP [26]. In that study, we found an increase in the number of infected samples and elevated serum levels of cytokines and total ROS associated with higher concentrations of O_2_. In the present study, we hypothesized that hyperoxia can cause specific organ damage and that this damage is correlated with ROS production.

## 2. Materials and Methods

### 2.1. Experimental Animals

We used male, pathogen-free, Sprague-Dawley rats of 12 to 13 weeks of age, weighing 285 ± 21 g (Charles River Laboratories, Barcelona, Spain). Animals were housed in the Animal Facilities of Hospital Universitario de Gran Canaria Dr. Negrin (Las Palmas, Spain) under standard care. We performed all experiments in accordance with the recommendations by the *Guide for the Care and Use of Laboratory Animals* [27], the guidelines of the Council of Europe (Europe Directive 2010/63/UE), and the Spanish Legislation for Laboratory Animal Welfare (RD 53/2013) under a protocol approved by the Ethics Committee for Experimentation and Animal Welfare of Hospital Universitario de Gran Canaria Dr. Negrin (CEEBA 003/2012). Animals received water and food *ad libitum*. At the end of the experimental period, animals were sacrificed by exsanguination after surgical cutting of the abdominal aorta under general anaesthesia. We followed ARRIVE guidelines for reporting animal research [28].

### 2.2. Sepsis Model and Experimental Groups

**Figure 1:**
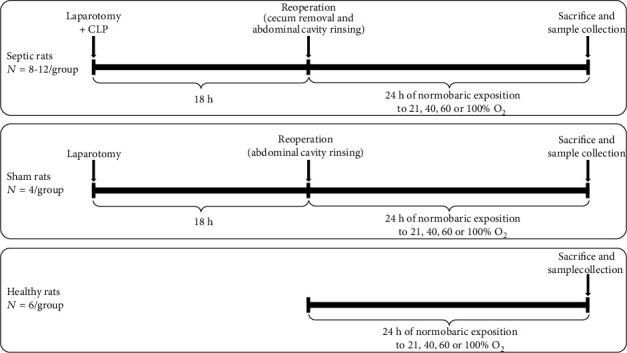
Schematic summary of experimental design and timeline. Septic and sham-septic animals underwent laparotomy (see text for details). At 18 h, after reopening the peritoneal cavity, removing the cecum in septic animals, and washing and closing the cavity, surviving septic, sham-septic, and healthy animals were randomly assigned to four groups and placed into a sealed Plexiglas cage continuously flushed with 21% (medical air), 40%, 60%, and 100% of oxygen. After 24 h, surviving animals were euthanized and samples were collected.

Sepsis was induced by CLP as previously described [29]. All procedures were performed under general anaesthesia, with a subcutaneous cocktail of fentanyl (Kern Pharma, Barcelona, Spain) and medetomidine (Esteve, Barcelona, Spain), both at 0.3 mg/kg. Briefly, after a laparotomy, the cecum was ligated and perforated twice, faeces were extruded, and the abdominal incision was closed. Eighteen hours later, the peritoneal cavity was reopened in surviving animals, the cecum removed, the cavity washed, and the abdomen closed. Animals received 10 mL of normal saline subcutaneously for fluid resuscitation. For a more detailed description of the protocol, see Supplementary Methods. This model closely mimics human sepsis by peritonitis. Sham-septic animals underwent the same surgical procedures, with the exception of CLP. We included an additional group of healthy rats as controls; these animals did not go through any surgery.  Figure 1 summarizes the timeline of the experimental design.

Immediately after the second surgical procedure, septic and sham-septic surviving animals and healthy rats were randomly assigned to four groups with different O_2_ concentrations, as previously described [26]. Briefly, animals were placed into a sealed Plexiglas cage continuously flushed with medical air (21% oxygen) or with mixed O_2_ reaching stable concentrations of 40%, 60%, and 100%. After 24 h of O_2_ exposure, surviving animals were euthanized. We studied a total of 12 experimental groups: CLP-septic animals breathing 21%, 40%, 60%, and 100% O_2_ (*n* = 12, 8, 9, and 10 animals, respectively), sham-septic animals breathing 21%, 40%, 60%, and 100% O_2_ (*n* = 4 animals per group), and healthy animals breathing 21%, 40%, 60%, and 100% O_2_ (*n* = 6 animals per group).

### 2.3. Blood and Tissue Sample Collection

At sacrifice, blood was drawn both from the jugular vein into EDTA K_3_ tubes and from the heart into separating tubes (Becton Dickinson) to obtain serum. Bronchoalveolar lavage fluid (BALF) was obtained in septic animals by insertion of a cannula into the trachea and careful injection and recovering of 5 mL of normal saline repeated four times. The right lung was removed and instilled with 10% buffered formalin and embedded in paraffin. The brain was also removed and infused with formalin and embedded in paraffin. Peritoneal fluid and urine were collected for microbiological analysis. In several animals, not all the samples were collected.

### 2.4. Histopathology

Sections of 4 *μ*m of lungs and brains were stained with haematoxylin-eosin and analysed by a pathologist blinded to group assignment. Lung inflammation and damage were semiquantitatively scored by using the following parameters: parenchymal disorganization, inflammatory infiltrates, alveolar rupture, alveolar oedema, and haemorrhage (on a scale from 0 (condition absent) to 4 (very severe)). The total lung pathology score was expressed as the sum of the scores of individual parameters. Brain damage was semiquantitatively scored based on the following parameters: inflammation, loss of normal brain architecture, neuronal damage, extramedullary haematopoiesis, and oedema (on a scale from 0 (condition absent) to 4 (very severe)). Brain damage scores were performed in samples from about half of the individuals in each group, because the other samples had been used for microbiological analysis. Immunostaining for glial fibrillary acidic protein (GFAP) was performed on brain sections to examine glial proliferation. After dewaxing and rehydration, antigen retrieval was performed at 95°C for 20 min in a Dako PT-link heater, using high pH antigen retrieval solution (Dako, Glostrup, Denmark). Then, endogenous peroxidase activity was inhibited with hydrogen peroxide solution (Dako), and slides were incubated with rabbit anti-GFAP (Dako) at room temperature in an Autostainer (Dako, CO, USA). Polymeric HRP-conjugated secondary antibody, DAB signal development, and haematoxylin counterstaining were also performed in an Autostainer, using commercial reagents and the manufacturer's protocol (all from Dako).

### 2.5. Assays

BALF was centrifuged, and the supernatant was collected in sterile tubes and stored at -80°C until measuring total proteins with an automated electrochemiluminescence immunoassay in a Cobas® 6000 analyser (Roche, Basel, Switzerland). The pellet was used to perform differential cell counts on Quick-Panoptic-stained smear preparations (modified Romanowsky staining) by direct counting under a light microscope. In several animals, BALF was not collected or the sample was damaged. Blood collected by cardiac puncture was centrifuged, and serum was collected and stored at -80°C. Interleukin-6 (IL-6) and soluble intercellular adhesion molecule- (sICAM-) 1 were measured using commercially available enzyme-linked immunosorbent assay kits (Abcam, Cambridge, UK, and MyBioSource, San Diego, CA, USA, respectively), and tumor necrosis factor-*α* (TNF-*α*) and IL-10 were measured using the CBA Flex Set cytometric bead array (BD Biosciences, Madrid, Spain). All these measurements were performed following the manufacturer's instructions. No sample was available for these measurements in several individuals. Troponin and creatine kinase (CK) levels were measured using an Elecsys® analyser (Roche, Basel, Switzerland). Levels of neuron-specific enolase (NSE), S100B, creatinine, urea, aspartate aminotransferase (ASAT), and alanine aminotransferase (ALAT) were measured using a Dimension EXL 200® analyser (Siemens Healthineers, Erlangen, Germany). Total ROS serum levels were measured using the Oxiselect in vitro assay kit (Cell Biolabs, San Diego, CA, USA) following the manufacturer's instructions [26].

### 2.6. Statistical Analysis

Data in tables are expressed as medians with quartiles 1 and 3 (Q1-Q3). Data in figures are expressed as box-and-whisker diagrams showing the smallest observation, lower quartile, median, upper quartile, and the largest observation. Comparisons among groups were performed using the Kruskal-Wallis test and between two independent groups using Mann–Whitney *U* test with Bonferroni correction for multiple comparisons when needed. We used the Jonckheere-Terpstra test for ordered differences in medians to test whether continuous variables had a trend with oxygen concentration. Correlations were assessed using Spearman's rho test. We did multiple linear regression, and due to the small sample size, it was supplemented with bootstrapping. For all analyses, we used SPSS Statistical Package version 15.0 (SPSS Inc., Chicago, IL, USA) and R version 3.5.3 (R Foundation for Statistical Computing, Vienna, Austria). For all comparisons, a two-sided *P* value < 0.05 was considered significant.

## 3. Results

Septic animals showed typical signs of systemic disease such as ruffled fur, lethargy, generalized weakness and reduced gross motor activity, and chromodacryorrhea. No differences in mortality rates were found in septic rats related to the oxygen group. All sham-septic and healthy animals survived the experimental period.

### 3.1. Effect of Oxygen Concentration on the Inflammatory Response

We found a significant linear trend of IL-6 with increasing oxygen concentrations in healthy animals (Table 1), as we had already observed in septic and sham-septic animals [26]. Two-by-two comparisons showed increased IL-6 levels in animals exposed to 100% O_2_ compared to other O_2_ concentrations, although after Bonferroni correction for multiple comparisons, differences in sham-septic animals did not remain statistically significant (Table 1). Healthy and septic animals exposed to 60% O_2_ had higher IL-6 levels than to 21% and 40% O_2_ (Table 1). In healthy animals, serum levels of TNF-*α* were under the detection limit, while IL-10 levels did not change among oxygen groups (data not shown). The serum levels of TNF-*α* and IL-10 in septic and sham-septic animals have been previously reported by our group [26].

**Figure 2:**
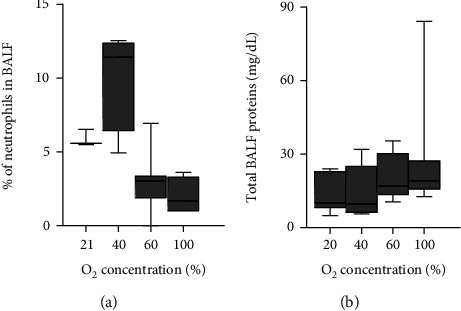
Neutrophils and total protein content in bronchoalveolar lavage fluid (BALF) from septic rats. Animals underwent cecal ligation and puncture (CLP) and were treated for 24 h with 21% (medical air), 40%, 60%, or 100% oxygen. No significant differences were found among groups (by Mann–Whitney test with Bonferroni correction). Data are box-and-whisker diagrams depicting the smallest observation, lower quartile, median, upper quartile, and largest observation. *n* = 3-7 per group for (a) and *n* = 7-9 per group for (b).

No relevant differences were found in the percentage of BALF neutrophils in relation to O_2_ concentration (Figure 2(a)). Although median BALF protein levels were higher in septic animals exposed to 60% and 100% O_2_, the differences among groups were not significant (Figure 2(b)).

### 3.2. Effect of Oxygen Concentration on Organ Injury

**Figure 3:**
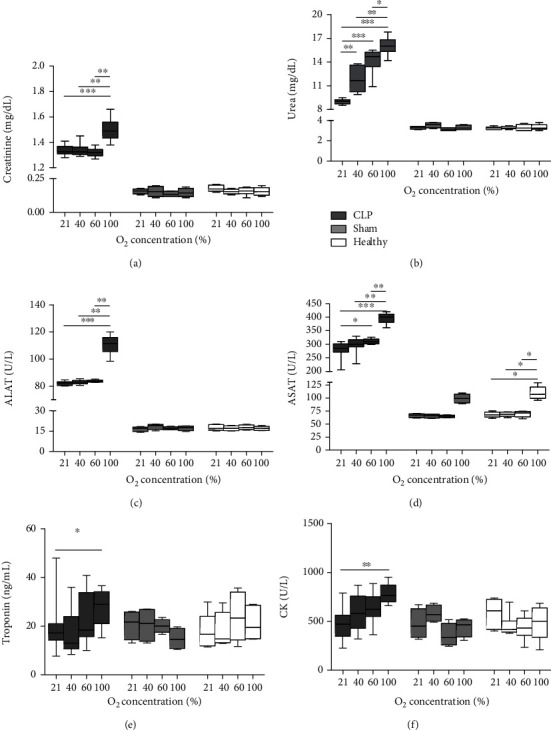
Biomarkers of organ damage in serum from septic, sham-septic, and healthy rats. Septic animals underwent cecal ligation and puncture (CLP). All animals were treated for 24 h with 21% (medical air), 40%, 60%, or 100% oxygen. Serum levels of creatinine (a), urea (b), ALAT (c), ASAT (d), troponin (e), and CK (f) are shown. Data are box-and-whisker diagrams depicting the smallest observation, lower quartile, median, upper quartile, and largest observation (*n* = 8-12 for septic, *n* = 4 for sham-septic, and *n* = 6 for healthy animals per group). ALAT: alanine transaminase; ASAT: aspartate aminotransferase; CK: creatine kinase. ^∗^*P* < 0.05, ^∗∗^*P* < 0.01, and ^∗∗∗^*P* < 0.001 by the Mann–Whitney *U* test with Bonferroni correction.

Septic animals had increased levels of creatinine and urea, with creatinine markedly augmented in animals exposed to 100% O_2_ and urea clearly increasing with increasing O_2_ concentration (*P* value for ordered differences in medians: 0.002 and <0.001, respectively). Both renal parameters remained low for all O_2_ concentrations in sham-septic and healthy rats (Figures 3(a) and 3(b)). The hepatocellular injury markers ALAT and ASAT increased in septic animals exposed to 100% O_2_ (Figures 3(c) and 3(d)) (*P* value for ordered differences in medians: <0.001 for both proteins). Of note, ASAT levels were also elevated in healthy (*P* = 0.001 for ordered differences in medians) and sham-septic animals exposed to 100% O_2_ (Figure 3(d)). In sham-septic animals, the differences were not statistically significant after Bonferroni correction. Serum troponin and CK were increased in septic animals exposed to 100% O_2_ compared to 21% O_2_ (Figures 3(e) and 3(f)) (*P* value for ordered differences in medians: 0.011 and <0.001, respectively).

**Figure 4:**
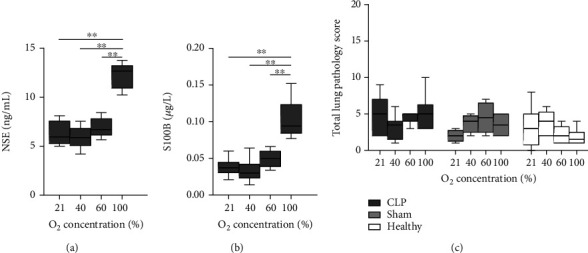
Brain and lung damage markers in septic, sham-septic, and healthy rats. Septic animals underwent cecal ligation and puncture (CLP), and all animals were treated for 24 h with 21% (medical air), 40%, 60%, or 100% oxygen. (a) Serum levels of neuron-specific enolase (NSE) in CLP rats (*n* = 8 per group); sham-septic and healthy animals had undetectable levels. (b) S100B serum levels in CLP rats (*n* = 8 per group); sham-septic and healthy animals had undetectable levels. (c) Semiquantitative histological total lung injury score in CLP (*n* = 8-10 per group), sham-septic (*n* = 4 per group), and healthy (*n* = 6 per group) rats. No significant differences were found among oxygen groups for the lung injury score. Data are box-and-whisker diagrams depicting the smallest observation, lower quartile, median, upper quartile, and largest observation. ^∗∗^*P* < 0.01 by the Mann–Whitney *U* test with Bonferroni correction.

Serum NSE and S100B were increased in septic rats receiving 100% O_2_ (Figures 4(a) and 4(b)) (*P* value for ordered differences in medians: <0.001 for both proteins). Healthy and sham-septic animals had undetectable values. Semiquantitative histology of brain tissue showed a nonsignificant increase of neuronal damage in septic animals exposed to 60 and 100% O_2_ compared to 21 and 40% O_2_ (Supplementary Figure S1). Only septic animals showed slight cerebral oedema, although unrelated to O_2_ concentration (Supplementary Figure S1). Similar findings occurred with GFAP (data not shown).

No significant differences were observed in histological lung pathology scores related to O_2_ concentrations in septic rats for any parameter of organ damage (Figure S2) nor for the total lung pathology score (Figure 4(c)). Septic animals had limited histological lung damage.

Serum levels of sICAM-1 were higher in septic animals than in sham-septic and healthy animals. Although the median level of sICAM-1 in septic animals exposed to 100% O_2_ was higher than in other O_2_ groups, the difference was not statistically significant (Figure S3). Some values in septic animals were above the detection limit, with the 100% O_2_ being the group with the highest number of samples over the cut-off point (50%).

### 3.3. Correlation between ROS Production and Biomarkers of Inflammation and Organ Damage

We found a moderated correlation between ROS and IL-6 in septic animals exposed to 100% O_2_ (*r* = 0.731, *P* = 0.040), but not for other O_2_ concentrations, nor when comparisons were made independent of O_2_. However, total IL-6 correlated significantly with most of the serum markers of organ damage measured in septic animals (Table S1).

### 3.4. Relation between Infection and Organ Damage

Serum urea levels of septic animals were significantly associated with positive culture in urine: 13.3 mg/dL (9.3-14.5) in animals with negative culture vs. 15.6 mg/dL (15.2-16.5) in animals with positive culture; *P* = 0.003. Although urea levels were also strongly associated with increasing O_2_ concentrations (see Section 3.2), a multivariate linear regression analysis showed that, when considering the combined effect of O_2_ concentration and positive urine culture, the variable that had a true effect on urea levels was the concentration of O_2_ (Table S2). As for creatinine, there was also a significant association with urine positive culture (1.3 mg/dL (1.3-1.7) vs. 1.5 mg/dL (1.4-1.5) in animals with negative and positive cultures, respectively; *P* = 0.017), but creatinine levels were only significantly increased in animals treated with 100% O_2_ (see Section 3.2). Multivariate linear regression analysis considering the combined effect of O_2_ concentration and positive urine culture showed that 100% O_2_, but not positive culture, had a true effect on creatinine levels (Table S3). We did not find any relation between infection and ROS levels.

## 4. Discussion

We had previously observed, in a clinically relevant model of sepsis by CLP, that the use of high concentrations of oxygen for 24 h increased the number of infected samples, as well as the serum levels of several cytokines and of ROS [26]. In the present study, we aimed to investigate, in the same preclinical model of sepsis, whether there was also an impact on organ damage and if it is related to ROS release. We found that the use of high O_2_ concentration increases the levels of IL-6 and several biomarkers of organ damage (creatinine, urea, ASAT, ALAT, troponin, CK, NSE, and S100B). Our findings suggest that, at least within the 24-hour study window, these effects were not related to ROS levels. This study lines up with previous reports suggesting that supplemental O_2_ should be used very cautiously in the management of septic patients due to the risk of enhanced inflammatory response and further deterioration of organ damage [22–25].

Studies on mouse models have reported an association between hyperoxia and lung damage as a result of inflammation and oxidative stress [30], cell death [15, 16], impairment of the surfactant system [15, 17], and increased lung injury and mortality in pulmonary infections [18, 19]. Hyperoxia caused a dose- and time-dependent inflammatory response in mechanically ventilated mice [20], and studies in critically ill patients have shown harmful effects of hyperoxia [22, 23]. Recent clinical trials have reported an association of hyperoxia with higher mortality, more episodes of shock, liver failure, and bacteraemia in critically ill patients [24] and increased rate of serious adverse events in patients with septic shock [25]. Serum IL-6 levels begin to rise 2 h after CLP, to decline >18 h after the surgical procedure, and its levels are predictors of the outcome [31]. In a previous study [26], we reported a gradual increase in IL-6 levels with increasing O_2_ concentration. In the present study, we found that although IL-6 serum levels were low in animals with no surgery, they followed the same trend as in septic animals in response to increasing O_2_ concentrations. This observation implies that hyperoxia not only exacerbates the inflammatory response caused by sepsis but also is detrimental by itself.

The lungs are the first organs receiving the impact of O_2_ therapy. However, in our model, we did not find relevant differences in BALF when comparing neutrophil cell counts or total protein among O_2_ groups. Also, analysis of lung pathology revealed no differences related to O_2_ concentration. We observed minimum lung damage in all groups, in line with previous studies describing no morphologic lung changes after 40 h of exposure to 100% O_2_ in rats [32] and only some moderate damage after 24 h in mice under 100% O_2_ [30]. In contrast to other models of acute lung injury, the CLP model has a slower onset and develops over days [33]. Our study was aimed at studying the response shortly after O_2_ therapy, and our findings suggest that for observing morphological lung damage, a wider time window could be necessary.

We examined the impact of O_2_ on extrapulmonary organs. Urea and ASAT gradually increased with increasing O_2_ concentration in septic animals, whereas creatinine and ALAT, which are more specific markers, were markedly higher in animals exposed to 100% O_2_. Unexpectedly, nonseptic animals under 100% O_2_ had elevated ASAT compared to the other oxygen groups (this was not significant in the sham-septic groups, probably due to smaller sample sizes). Based on our findings, we postulate that high O_2_ concentrations enhance renal and hepatic damage induced by sepsis and that administration of 100% O_2_ can also be deleterious in healthy individuals. Previous studies in mouse models of CLP-induced sepsis [34] and zymosan-induced inflammation [6, 35] showed that exposure to 98-100% O_2_ for 3 h resulted in lower levels of biomarkers for kidney and liver function than in mice exposed to room air. The differences of those studies with our study are likely due to shorter periods of O_2_ treatment in those studies. Since those animals were not sacrificed right after the O_2_ exposure, but 15-20 h later [6, 34, 35], the differences observed with our study would point to differences in exposure time and not in the time elapsed since the initiation of O_2_ therapy. Cardiac dysfunction occurs during the early stages of sepsis. In our study, biomarkers of cardiac injury increased significantly in septic animals exposed to high O_2_ concentration, although they were not as marked as renal and hepatic scores. Of note, troponin and CK levels were not as dramatically increased in septic compared to nonseptic animals than other markers of damage. The brain is often affected very early in the course of sepsis. In our study, serum NSE and S100B were markedly elevated in septic animals exposed to 100% O_2_. However, histopathological brain analysis did not show relevant damage, nor significant association with O_2_ administration. As for lung pathology, it is plausible that the time elapsed after CLP and O_2_ exposure was not sufficient to see morphological changes in cerebral tissue, but enough for the release of activation/damage markers from the brain into the blood. Also, it could be too early to see significant differences in circulating ICAM-1 (an indicator of endothelial damage) among O_2_ groups, despite the fact that it was higher in septic than in nonseptic animals. High sICAM-1 levels in BALF have been observed in healthy mice under hyperoxia but only after 3 days of exposure to 95% O_2_ [36].

Since we had previously reported elevated total ROS levels associated with supplemental O_2_ [26], we investigated whether ROS levels correlate with biomarkers of inflammation and organ injury in septic animals. Although IL-6 is strongly correlated with several serum markers of organ damage, no relevant correlation was found for serum ROS levels with any of them. Unfortunately, we did not measure ROS in healthy animals, but since ROS levels were undetectable in sham-septic rats, it is plausible that ROS levels are also undetectable in healthy controls. Nevertheless, IL-6 and ASAT were elevated in healthy rats under hyperoxia. All these findings point to an inflammatory mediation in organ damage in which ROS production is not directly involved. However, a role for ROS in shorter or longer exposure to hyperoxia cannot be ruled out [30]. We also reported [26] an effect of O_2_ on the number of samples with positive cultures. The gradual increase of infected samples related to O_2_ concentration was especially clear in urine samples. In the current study, we went a step further examining the combined effects of O_2_ and positive urine culture on renal damage and found that the variable that had a true effect was O_2_ concentration, not the infection.

We acknowledge some limitations to our study. First is the small sample size of the sham-septic and control groups, which makes it difficult to achieve statistical significance, although pointing to clinical or physiological relevance. Second, although we used a clinically relevant preclinical model, it is an experimental model, and we cannot definitively extrapolate our findings to the ICU setting, where patients have multiple causes of sepsis, comorbidities, and unpredicted progression of their underlying disease. Third is the use of a unique and early time point. Since the aim of our study was to analyse the short-term inflammatory damage mediated by O_2_ administration, we performed our analyses after 24 h of O_2_ therapy. As previously discussed, this time seems to be insufficient to observe macroscopic tissue damage and does not allow us to assert what happens later. However, it does allow us to confirm that, after 24 h of hyperoxia, several organs were damaged, as manifested by a marked increase in levels of organ damage biomarkers, and also to propose that this damage is independent of ROS release. Based on our observations, we could speculate that macroscopic tissue damage would be visible later, although further studies using longer time points are needed. Our findings highlight the risks associated with the routine practice of treating critically ill patients with high O_2_ concentrations. The use of liberal O_2_ therapy is not recommended as initially thought. The use of supplemental O_2_ in critically ill patients should be carefully adjusted to achieve a proper balance between beneficial and potentially detrimental effects [37]. Long-term effects of O_2_ therapy still need to be assessed prospectively in homogeneous cohorts in clinical trials, and further studies are needed to address the underlying molecular mechanisms of organ damage.

In conclusion, administration of high concentrations of O_2_ is associated with enhanced inflammatory response and increased markers of multiple organ damage in a clinically relevant preclinical model of intra-abdominal sepsis. In addition, our results do not support a direct role of ROS release in this injurious effect. Our findings add relevant data to the controversy about deleterious effects of hyperoxia. Even though the use of supplemental O_2_ is a common therapeutic intervention in septic patients, it may also induce pathological effects. The results of the present study support that the use of a liberal O_2_ therapy is not as recommendable as initially thought and highlight the need for developing safer guidelines for O_2_ therapy.

## Figures and Tables

**Table 1:** Serum IL-6 levels in healthy, sham-septic, and CLP-septic rats at the different oxygen concentrations.

	21% O_2_	40% O_2_	60% O_2_	100% O_2_	*P* ^∗^	*P* ^∗∗^
Healthy *n* = 6/group	15.8^###^ (14.2-21.0)	19.5^###^ (16.2-23.0)	39.0 (29.9-42.2)	55.7 (41.9-59.8)	<0.001	<0.001
Sham-septic *n* = 4/group	49.2 (44.2-54.9)	51.1 (45.7-55.7)	58.4 (51.5-65.1)	85.8 (82.3-87.8)	0.020	0.001
CLP *n* = 8-10/group	399.5^##§^ (328.5-454.2)	425.7^§^ (413.8-472.4)	554.9^§^ (516.7-570.7)	724.0 (649.9-744.0)	<0.001	<0.001

Values are expressed in pg/mL, as median (Q1-Q3). CLP: cecal ligation and puncture. ^∗^*P* value of the comparison between the four groups determined by the Kruskal-Wallis test. ^∗∗^*P* value of the tendency among the four groups determined by the Jonckheere-Terpstra test for ordered differences. ^#^*P* < 0.05 determined by the Mann–Whitney *U* test with Bonferroni correction when compared to 100%. ^##^*P* < 0.05 determined by the Mann–Whitney *U* test with Bonferroni correction when compared to 60%. ^§^*P* < 0.01 determined by the Mann–Whitney *U* test with Bonferroni correction when compared to 100%.

## Data Availability

Data used to support the findings of this study are available from the corresponding author upon request.

## Supplementary Materials

Supplementary Materials This file includes supplementary data: supplementary tables (Tables S1–S3) and figures (Figures S1–S3) and supplementary methods: a detailed description of the CLP protocol.

## References

[1] Singer M., Deutschman C. S., Seymour C. W. (2016). The third international consensus definitions for sepsis and septic shock (sepsis-3). *Journal of the American Medical Association*.

[2] Blanco J., Muriel-Bombín A., Sagredo V. (2008). Incidence, organ dysfunction and mortality in severe sepsis: a Spanish multicentre study. *Critical Care*.

[3] Prescott H. C., Costa D. K. (2018). Improving long-term outcomes after sepsis. *Critical Care Clinics*.

[4] Vincent J.-L., Moreno R., Takala J. (1996). The SOFA (sepsis-related organ failure assessment) score to describe organ dysfunction/failure. *Intensive Care Medicine*.

[5] Barth E., Bassi G., Maybauer D. M. (2008). Effects of ventilation with 100% oxygen during early hyperdynamic porcine fecal peritonitis. *Critical Care Medicine*.

[6] Hou L., Xie K., Qin M. (2010). Effects of reactive oxygen species scavenger on the protective action of 100% oxygen treatment against sterile inflammation in mice. *Shock*.

[7] Hauser B., Barth E., Bassi G. (2009). Hemodynamic, metabolic, and organ function effects of pure oxygen ventilation during established fecal peritonitis-induced septic shock. *Critical Care Medicine*.

[8] Calzia E., Asfar P., Hauser B. (2010). Hyperoxia may be beneficial. *Critical Care Medicine*.

[9] Asfar P., Calzia E., Huber-Lang M., Ignatius A., Radermacher P. (2012). Hyperoxia during septic shock-Dr. Jekyll or Mr. Hyde?. *Shock*.

[10] Waisman D., Brod V., Rahat M. A. (2012). Dose-related effects of hyperoxia on the lung inflammatory response in septic rats. *Shock*.

[11] He X., Su F., Xie K., Taccone F. S., Donadello K., Vincent J. L. (2017). Should hyperoxia be avoided during sepsis? An experimental study in ovine peritonitis. *Critical Care Medicine*.

[12] Gore A., Muralidhar M., Espey M. G., Degenhardt K., Mantell L. L. (2010). Hyperoxia sensing: from molecular mechanisms to significance in disease. *Journal of Immunotoxicology*.

[13] Sies H. (1997). Oxidative stress: oxidants and antioxidants. *Experimental Physiology*.

[14] Lee P. J., Choi A. M. (2003). Pathways of cell signaling in hyperoxia. *Free Radical Biology & Medicine*.

[15] Shimada I., Kubota A., Katoh M., Suzuki F. (2016). Hyperoxia causes diffuse alveolar damage through mechanisms involving upregulation of *c-Myc/Bax* and enhanced production of reactive oxygen species. *Respiratory Investigation*.

[16] Makena P. S., Luellen C. L., Balazs L. (2010). Preexposure to hyperoxia causes increased lung injury and epithelial apoptosis in mice ventilated with high tidal volumes. *American Journal of Physiology-Lung Cellular and Molecular Physiology*.

[17] Schwingshackl A., Lopez B., Teng B. (2017). Hyperoxia treatment of TREK-1/TREK-2/TRAAK-deficient mice is associated with a reduction in surfactant proteins. *American Journal of Physiology. Lung Cellular and Molecular Physiology*.

[18] Tateda K., Deng J. C., Moore T. A. (2003). Hyperoxia mediates acute lung injury and increased lethality in Murine *Legionella* Pneumonia: the role of apoptosis. *Journal of Immunology*.

[19] Kikuchi Y., Tateda K., Fuse E. T. (2009). Hyperoxia exaggerates bacterial dissemination and lethality in Pseudomonas aeruginosa pneumonia. *Pulmonary Pharmacology & Therapeutics*.

[20] Helmerhorst H. J. F., Schouten L. R. A., Wagenaar G. T. M. (2017). Hyperoxia provokes a time- and dose-dependent inflammatory response in mechanically ventilated mice, irrespective of tidal volumes. *Intensive Care Medicine Experimental*.

[21] Damiani E., Donati A., Girardis M. (2018). Oxygen in the critically ill: friend or foe?. *Current Opinion in Anaesthesiology*.

[22] Stolmeijer R., Bouma H. R., Zijlstra J. G., Drost-de Klerck A. M., Ter Maaten J. C., Ligtenberg J. J. M. (2018). A systematic review of the effects of hyperoxia in acutely ill patients: should we aim for less?. *BioMed Research International*.

[23] Vincent J. L., Taccone F. S., He X. (2017). Harmful effects of hyperoxia in postcardiac arrest, sepsis, traumatic brain injury, or stroke: the importance of individualized oxygen therapy in critically ill patients. *Canadian Respiratory Journal*.

[24] Girardis M., Busani S., Damiani E. (2016). Effect of conservative vs conventional oxygen therapy on mortality among patients in an intensive care unit: the oxygen-ICU randomized clinical trial. *JAMA*.

[25] Asfar P., Schortgen F., Boisramé-Helms J. (2017). Hyperoxia and hypertonic saline in patients with septic shock (HYPERS2S): a two-by-two factorial, multicentre, randomised, clinical trial. *The Lancet Respiratory Medicine*.

[26] Rodríguez-González R., Martín-Barrasa J. L., Ramos-Nuez Á. (2014). Multiple system organ response induced by hyperoxia in a clinically relevant animal model of sepsis. *Shock*.

[27] National Research Council (1996). *Guide for the Care and Use of Laboratory Animals*.

[28] Kilkenny C., Browne W. J., Cuthill I. C., Emerson M., Altman D. G. (2010). Improving bioscience research reporting: the ARRIVE guidelines for reporting animal research. *PLoS Biology*.

[29] Villar J., Ribeiro S. P., Mullen J. B., Kuliszewski M., Post M., Slutsky A. S. (1994). Induction of the heat shock response reduces mortality rate and organ damage in a sepsis-induced acute lung injury model. *Critical Care Medicine*.

[30] Nagato A. C., Bezerra F. S., Lanzetti M. (2012). Time course of inflammation, oxidative stress and tissue damage induced by hyperoxia in mouse lungs. *International Journal of Experimental Pathology*.

[31] Gao M., Zhang L., Liu Y. (2012). Use of blood urea nitrogen, creatinine, interleukin-6, granulocyte-macrophage colony stimulating factor in combination to predict the severity and outcome of abdominal sepsis in rats. *Inflammation Research*.

[32] Crapo J. D., Barry B. E., Foscue H. A., Shelburne J. (1980). Structural and biochemical changes in rat lungs occurring during exposures to lethal and adaptive doses of oxygen. *The American Review of Respiratory Disease*.

[33] Matute-Bello G., Frevert C. W., Martin T. R. (2008). Animal models of acute lung injury. *American Journal of Physiology. Lung Cellular and Molecular Physiology*.

[34] Xie K., Fu W., Xing W. (2012). Combination therapy with molecular hydrogen and hyperoxia in a murine model of polymicrobial sepsis. *Shock*.

[35] Hong Y., Sun L., Sun R., Chen H., Yu Y., Xie K. (2016). Combination therapy of molecular hydrogen and hyperoxia improves survival rate and organ damage in a zymosan-induced generalized inflammation model. *Experimental and Therapeutic Medicine*.

[36] Mendez M. P., Morris S. B., Wilcoxen S., Greeson E., Moore B., Paine R. (2006). Shedding of soluble ICAM-1 into the alveolar space in murine models of acute lung injury. *American Journal of Physiology. Lung Cellular and Molecular Physiology*.

[37] Villar J., Kacmarek R. M. (2017). Oxygen: breath of life or kiss of death. *Critical Care Medicine*.

