# Supplementary material for: Systemic Effects Induced by Hyperoxia in a Preclinical Model of Intra-abdominal Sepsis
Source: Mediators Inflamm. 2020 Oct 15;2020:5101834. doi: 10.1155/2020/5101834 (PMC7585649; doi:10.1155/2020/5101834)
Supplement: Supplementary Materials — This file includes supplementary data: supplementary tables (Tables S1–S3) and figures (Figures S1–S3) and supplementary methods: a detailed description of the CLP protocol. [file 5101834.f1.pdf]

SUPPLEMENTARY MATERIAL

Systemic Effects Induced by Hyperoxia in a Preclinical Model of Intraabdominal Sepsis

M. Isabel García-Laorden[1, 2], Raquel Rodríguez-González[3, 4], José L. Martín-Barrasa[2, 5], Sonia García-Hernández[6], Ángela Ramos-Nuez[1, 2], H. Celeste González-García[6], Jesús M. González-Martín[2], Robert M. Kacmarek[7, 8] and Jesús Villar[1, 2, 9]

[1]CIBER de Enfermedades Respiratorias, Instituto de Salud Carlos III, Monforte de Lemos 3-5, Pabellón 11, 28029 Madrid, Spain.

[2]Multidisciplinary Organ Dysfunction Evaluation Research Network, Research Unit, Hospital Universitario de Gran Canaria Dr. Negrín, Barranco de la Ballena s/n, 35019 Las Palmas de Gran Canaria, Spain.

[3]Department of Psychiatry, Radiology, Public Health, Nursing and Medicine, School of Nursing, University of Santiago de Compostela, Avda. Xoán XXIII s/n, 15782 Santiago de Compostela, Spain.

[4]Department of Anaesthesiology, Critical Care and Pain Management, Hospital Clínico Universitario, Health Research Institute of Santiago de Compostela (IDIS), Travesa da Choupana s/n, 15706 Santiago de Compostela, Spain.

[5]Animal Infectious Diseases and Ictiopathology, University Institute of Animal Health (IUSA), University of Las Palmas de Gran Canaria, Carretera de Trasmontaña s/n, 35416 Arucas, Spain.

[6]Department of Pathology, Hospital Universitario de Canarias, Carretera Cuesta Taco 0, 38320 Sta. Cruz de Tenerife, Spain.

[7]Department of Respiratory Care, Massachusetts General Hospital, 55 Fruit Street, Boston, MA 02114, USA.

[8]Department of Anaesthesiology, Harvard University, 55 Fruit Street, Boston, MA 02114,USA.

[9] Keenan Research Center for Biomedical Science at the Li Ka Shing Knowledge Institute, St. Michael's Hospital, 209 Victoria Street, Toronto, ON M5B1T8, Canada.

Correspondence: Dr. Jesús Villar; jesus.villar54@gmail.com

Supplementary Data

Table S1. Correlation between serum levels of IL-6 and organ damage biomarkers in septic-CLP rats.

	IL-6		
	r	***P***	***n***
Creatinine	0.548	0.001	35
Urea	0.755	<0.001	35
ALAT	0.582	<0.001	35
ASAT	0.964	<0.001	35
Troponin	0.313	0.067	35
CK	0.438	0.009	35
NSE	0.680	<0.001	29
S100B	0.713	<0.001	29

r: Spearman's rho coefficient of correlation. CLP: cecal ligation and puncture. ALAT: alanine aminotransferase; ASAT: aspartate aminotransferase; CK: creatine kinase; NSE: neuron-specific enolase.

Table S2. Multiple linear regression analysis of the variables urine positive culture and oxygen percentage related to urea serum levels in septic-CLP rats.

	Multiple linear regression				BOOTSTRAPPING	
**Variables**	**b**	**Se**	***P*-value**	**IC (95%)**	**b**	**IC (95%)**
Constant	8.89	0.48	< 0.001	7.89 – 9.88	8.89	8.68 – 9.21
Urine positive culture: YES	0.36	0.59	0.55	-0.86 –1.58	0.34	-1.41 – 1.66
Oxygen: 21%	Ref.	-	-	-	-	-
Oxygen: 40%	2.91	0.67	0.0002	1.54 – 4.27	2.87	1.67 – 4.07
Oxygen: 60%	5.17	0.66	< 0.001	3.82 – 6.52	5.17	3.78 – 5.99
Oxygen: 100%	7.04	0.73	< 0.001	5.55 – 8.52	7.1	5.93 – 8.57

R^2= 0.83. CLP: cecal ligation and puncture.

Table S3. Multiple linear regression analysis of the variables urine positive culture and oxygen percentage related to creatinine serum levels in septic-CLP rats.

	Multiple linear regression				BOOTSTRAPPING	
**Variables**	**b**	**Se**	***P*-value**	**IC (95%)**	**b**	**IC (95%)**
Constant	1.34	0.02	< 0.001	1.30-1.39	1.34	1.31-1.37
Urine positive culture: YES	0.02	0.02	0.442	-0.04-0.08	0.02	-0.08-0.09
Oxygen: 21%	Ref.	-	-	-	-	-
Oxygen: 40%	-0.005	0.03	0.86	-0.07-0.06	-0.003	-0.04-0.05
Oxygen: 60%	-0.03	0.03	0.40	-0.09-0.04	-0.03	-0.07-0.01
Oxygen: 100%	0.16	0.03	< 0.001	0.09-0.23	0.16	0.09-0.25

R^2= 0.64. CLP: cecal ligation and puncture.

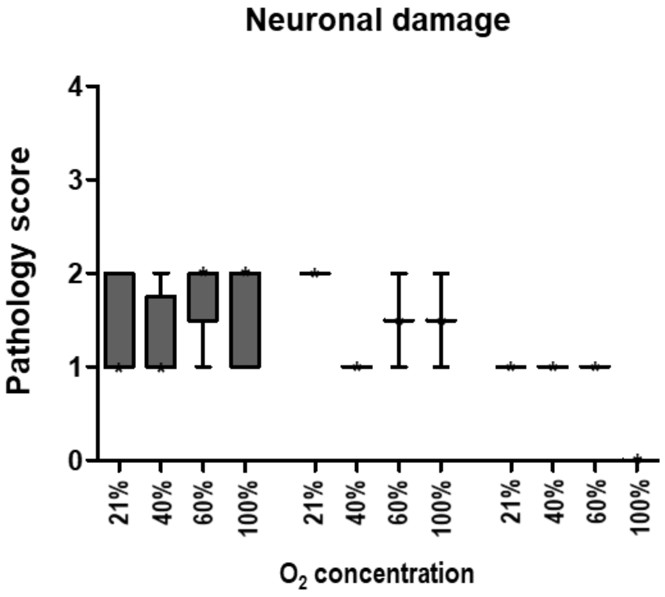

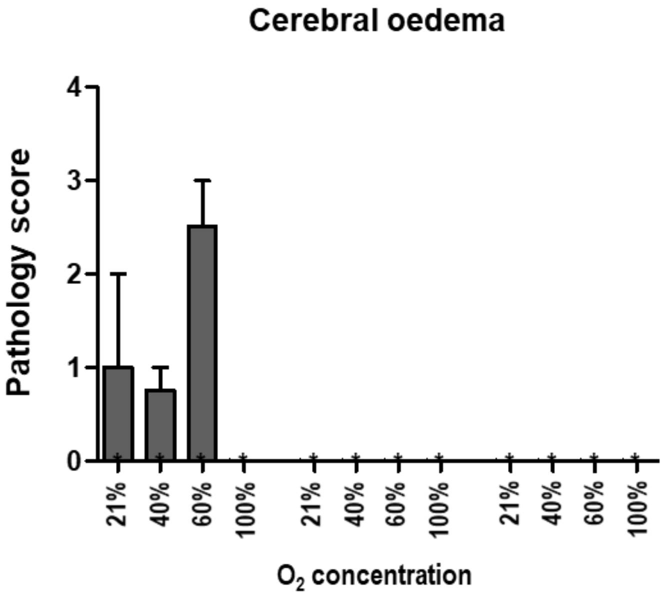

Figure S1. Semi-quantitative histology injury scores of brain in septic, sham-septic and healthy rats. Septic animals underwent cecal ligation and puncture (CLP), and all animals were exposed to 21, 40, 60 or 100% oxygen for 24h. No significant differences were found in the comparisons between oxygen groups for the brain injury scores. Data are box-and-whisker diagrams depicting the smallest observation, lower quartile, median (highlighted as *), upper quartile, and largest observation. N= 4-5 per group in septic, 2 per group in sham-septic and 3 per group in healthy rats.

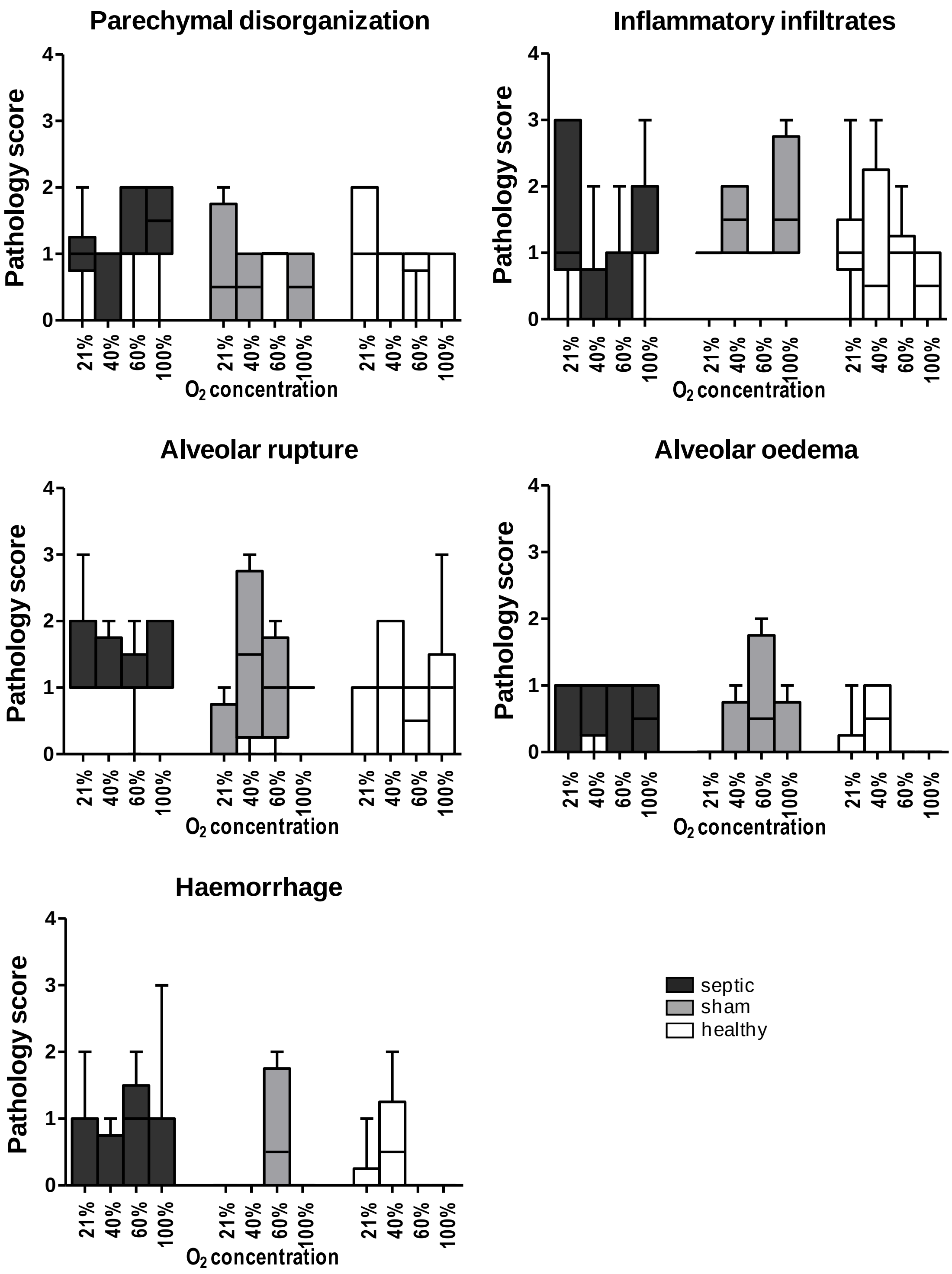

Figure S2. Semi-quantitative histology injury scores of lungs in septic, sham-septic and healthy rats. Septic animals underwent cecal ligation and puncture (CLP), and all animals were exposed to 21 (medical air), 40, 60 or 100% oxygen for 24h. No significant differences were found in the comparisons between oxygen groups for the lung injury scores. Data are box-and-whisker diagrams depicting the smallest observation, lower quartile, median, upper quartile, and largest observation. N= 8-10 per group in septic, 4 per group in sham-septic and 6 per group in healthy rats.

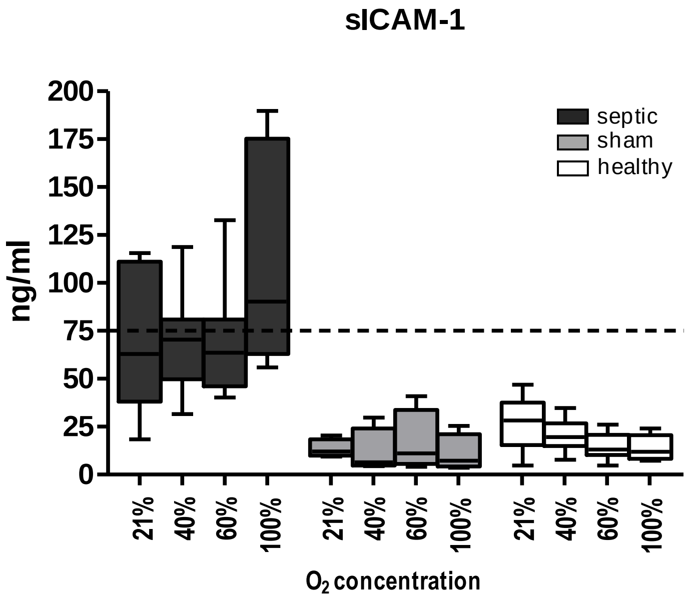

Figure S3. Serum levels of sICAM-1 in septic, sham-septic and healthy rats. Septic animals underwent cecal ligation and puncture (CLP), and all animals were exposed to 21 (medical air), 40, 60 or 100% oxygen for 24h. No significant differences were found in the comparisons between oxygen groups. Data are box-and-whisker diagrams depicting the smallest observation, lower quartile, median, upper quartile, and largest observation. Dashed line represents the upper detection limit of sICAM-1. N= 7-8 per group in septic, 4 per group in sham-septic and 6 per group in healthy rats.

Supplementary Methods

Sepsis model

Sepsis was induced by cecal ligation and puncture (CLP). This study used the methods previously described by Villar *et al* [1] and Rittirsch *et al* [2], and its description partly reproduces their wording.

With the animals under general anaesthesia (a subcutaneous cocktail of medetomidine [Esteve, Barcelona, Spain] / fentanyl [KERN Pharma, Barcelona, Spain] at a dose of 0.3/0.3 mg/kg) and breathing spontaneously, a laparotomy through a midline abdominal incision was performed. Next, the cecum was ligated below the ileocecal valve in such a position that the ratio between the ligated cecum (distance between the distal pole and the ligation) and the distance from the ligation to the basis of the cecum (immediately below the ileocecal valve) is consistent with mid-grade sepsis. Then, using an 18G needle, the cecum was perforated in two locations, approximately 1 cm apart, midway between the ligation and the tip of the cecum in a mesenteric-to-antimesenteric direction. Faeces were extruded by gentle compression of the cecum. The bowel was returned to the abdomen and the incision was closed by applying simple running sutures to the abdominal musculature and metallic clips to the skin. Animals were observed in a recovery cage until they awake. Eighteen hours after the CLP procedure, with the animals under general anaesthesia and breathing spontaneously, the peritoneal cavity was reopened. The cecum was excised distal to the ligature and removed, the cavity was washed with 10 mL of warm, normal saline, and the abdomen was closed. Animals received 10mL of normal saline subcutaneously for fluid resuscitation. Although broad-spectrum antibiotic therapy has been used resulting in improved survival [2], we decided not to administer antibiotics since the

interest of our study was not only simulating the clinical picture of sepsis, but also investigating its basic pathogenesis.

CLP is a widely used model which closely mimics human sepsis caused by peritonitis. We introduced a modification, a post-CLP laparotomy followed by excision of the necrotic cecum and peritoneal washing. This intervention was previously shown by Rittirsch *et al* [2] to simulate abdominal sepsis and its treatment in human individuals, that is, to mimic the clinical situation. Despite the timing of damage control surgery (gastrointestinal reconstruction and closure of the abdominal cavity) is discussed intensively in the literature, the classical surgical source control (debridement, removal of infected devices, drainage of purulent cavities, and decompression of the abdominal cavity) is the gold standard of surgical care in case of intra-abdominal sepsis [3].

Supplementary Methods References

1. Villar J, Ribeiro SP, Mullen JB, Kuliszewski M, Post M, Slutsky AS. Induction of the heat shock response reduces mortality rate and organ damage in a sepsis-induced acute lung injury model. Crit Care Med. 1994 Jun;22(6):914-21.

2. Rittirsch D, Huber-Lang MS, Flierl MA, Ward PA. Immunodesign of experimental sepsis by cecal ligation and puncture. Nat Protoc. 2009;4(1):31-6. doi: 10.1038/nprot.2008.214.

3. Hecker A, Reichert M, Reuß CJ, Schmoch T, Riedel JG, Schneck E, Padberg W, Weigand MA, Hecker M. Intra-abdominal sepsis: new definitions and current clinical standards. Langenbecks Arch Surg. 2019;404(3):257-271. doi: 10.1007/s00423-019-01752-7.
